# Severity Level and Duration of Energy Deficit in Mice Affect Bone Phenotype and Bone Marrow Stromal Cell Differentiation Capacity

**DOI:** 10.3389/fendo.2022.880503

**Published:** 2022-06-06

**Authors:** Viktorija Avilkina, Damien Leterme, Guillaume Falgayrac, Jérôme Delattre, Flore Miellot, Véronique Gauthier, Christophe Chauveau, Olfa Ghali Mhenni

**Affiliations:** ^1^ MAB Lab ULR4490, Univ Littoral Côte d’Opale, Boulogne-sur-Mer, France; ^2^ MAB Lab ULR4490, Univ Lille Nord de France, Lille, France

**Keywords:** energy deficit, bone, bone marrow adiposity, sirtuin type 1 (SIRT1), bone marrow stromal cell (BMSC), differentiation

## Abstract

Anorexia nervosa is known to induce changes in bone parameters and an increase in bone marrow adiposity (BMA) that depend on the duration and seriousness of the disease. Previous studies have found that bone loss is associated with BMA accumulation. Sirtuin of type 1 (Sirt1), a histone deacetylase that is partly regulated by energy balance, was shown to have pro-osteoblastogenic and anti-adipogenic effects. To study the effects of the severity and duration of energy deficits related to bone loss, a mouse model of separation-based anorexia (SBA) was established. We recently demonstrated that moderate body weight loss (18%) 8-week SBA protocol in mice resulted in an increase in BMA, bone loss, and a significant reduction in Sirt1 expression in bone marrow stromal cells (BMSCs) extracted from SBA mice. We hypothesised that Sirt1 deficit in BMSCs is associated with bone and BMA alterations and could potentially depend on the severity of weight loss and the length of SBA protocol. We studied bone parameters, BMA, BMSC differentiation capacity, and Sirt1 expression after induction of 4 different levels of body weight loss (0%,12%,18%,24%), after 4 or 10 weeks of the SBA protocol. Our results demonstrated that 10 week SBA protocols associated with body weight loss (12%, 18%, 24%) induced a significant decrease in bone parameters without any increase in BMA. BMSCs extracted from 12% and 18% SBA groups showed a significant decrease in Sirt1 mRNA levels before and after co-differentiation. For these two groups, decrease in Sirt1 was associated with a significant increase in the mRNA level of adipogenic markers and a reduction of osteoblastogenesis. Inducing an 18% body weight loss, we tested a short SBA protocol (4-week). We demonstrated that a 4-week SBA protocol caused a significant decrease in Tb.Th only, without change in other bone parameters, BMA, Sirt1 expression, or differentiation capacity of BMSCs. In conclusion, this study showed, for the first time, that the duration and severity of energy deficits are critical for changes in bone parameters, BMSC differentiation, and Sirt1 expression. Furthermore, we showed that in this context, Sirt1 expression could impact BMSC differentiation with further effects on bone phenotype.

## Introduction

Anorexia nervosa (AN) is a disorder with psychiatric origins characterized by inadequate dietary intake resulting in low body weight. Many studies have demonstrated that a higher volume of bone marrow adipose tissue (BMAT) is present in anorexic patients than in normal-weight controls (1–3). This high level of BMAT is often associated with a decline in bone mass, resulting in increased fracture risk (4, 5). Recently, a negative correlation was shown between the fraction of fat in the bone marrow cavity and the bone mineral density (BMD) at the femoral neck and at the hip of anorexic patients in multiple anatomical subregions, even after weight recovery (6).

Marrow adipocytes originate from skeletal stem cells (SSCs) that also function as precursors to muscle, cartilage, and bone-forming osteoblasts (7–9). Moreover, it has been shown that the accumulation of marrow adipocytes, which is observed in bone loss situations, is potentially caused by a shift in the commitment of SSCs from the osteogenic pathway to the adipogenic pathway (10). This finding suggested the existence of a competitive relationship between these two pathways. Furthermore, bone marrow stromal cells (BMSCs), which include SSCs from aged mice express more adipocytic transcripts and fewer osteoblastic transcripts than BMSCs extracted from young animals (11).

The differentiation of SSCs towards the adipocytic or osteoblastic pathway could be influenced by many modulators of signalling pathways, such as glucocorticoids, Wnts, and bone morphogenetic proteins (12–14). Moreover, the histone deacetylase (HDAC) family was also shown to influence the fate of differentiating mesenchymal progenitor cells by removing acetyl groups from lysine residues in histones and other proteins, thus, altering chromatin structure, gene expression, and protein activity (15, 16).

Sirtuin type 1 (Sirt1) is classified as a class III HDAC and is known to be involved in the extension of lifespan in mammals and other organisms and in protection against age-associated diseases (such as diabetes and obesity) (17). Additionally, Sirt1 is involved in the regulation of BMSC differentiation into either osteoblasts or adipocytes. Indeed, several studies have demonstrated that activation of Sirt1 promotes osteoblastic differentiation (18–21), while a decrease in Sirt1 expression induces adipocytic differentiation (22, 23). Interestingly, caloric restriction (CR) has been shown to stimulate the expression and activity of Sirt1 in various mammalian tissues, such as liver and white adipose tissue. Also, CR has been shown to induce a decrease in the level of Sirt1 mRNA in the cerebellum and midbrain (24, 25).

Recently, we demonstrated that 8 weeks of a separation-based anorexia (SBA) protocol, inducing medium weight loss (18%) vs day 0 of experiment in mice, is associated with an increase in bone marrow adiposity (BMA), bone loss, and a significant reduction in Sirt1 expression in BMSCs extracted from SBA mice (26). We hypothesised that Sirt1 deficit in BMSCs is involved in bone and BMA alterations and could potentially depend on the severity of weight loss and the length of SBA protocol. To verify these hypotheses, we characterized bone and BMA, and determined BMSC differentiation capacity and Sirt1 expression after a 10-week induction of 4 different levels of body weight changes (0%, -12%, -18% and -24%) relative to mice at Day 0. The same parameters were also analyzed after a 4-week SBA protocol with 18% weight loss.

## Materials and Methods

### Animals

Seven-week-old female C57BL/6J mice purchased from Charles River Laboratories (St Germain sur l’Abresle, France) were used for the separation-based anorexia (SBA) model (27). This particular mouse strain was chosen because of its use in various studies related to calorie restriction-induced bone alterations. Additionally, C57BL/6J mice exhibit a medium level of BMA, which allows us to detect any change in this parameter.

Mice were housed 6 per cage in a controlled room temperature (22°C+/- 1°C) under a 12-hour dark/light cycle (lights off at 10 a.m.) and with free access to water. Before induction of the SBA protocol, mice were acclimatized for one week. The SBA protocol was generated for ten weeks and aimed to induce 4 different weight loss conditions in randomly allocated mice – no weight loss (SBA 0%), a light 12% weight loss (SBA 12%) (achieved 12.97%), a moderate body weight loss of 18% (SBA 18%) (achieved 17.27%) and severe weight loss of 24% (SBA 24%) (achieved 23.17%), relative to Day 0 (D0). A time restricted-feeding was used to induce weight loss in mice, a food access time was gradually reduced from 6 h to 2 h per day during the protocol, depending on the desired weight loss to achieve. Mice allocated to severe, moderate, and light weight loss groups had reduced food availability and time dependent on the progression of weight loss. For no weight loss group the time of the feeding was not decreased dramatically, in order to maintain the initial mice weight. Furthermore, throughout the protocol mice of SBA groups were kept in the separate sections of the cage with free access to water and this encouraged the increased energy expenditure by thermogenesis. The SBA mice were gathered together in regular cages for the periods of feeding. The CT group was housed in standard conditions in collective cages with water and food provided *ad libitum*. This study was specifically approved by the Committee on the Ethics of Animal Experiments (CEEA) of Nord-Pas de Calais, France (permit number: CEEA#2016070717275082).

Additionally, a 4-week SBA protocol was performed using seven-week-old female C57BL/6J mice, similar to the 10-week protocol. After one week of acclimatization, the SBA protocol was generated for 4 weeks to achieve moderate weight loss of 18% (SBA 18%) (achieved 18.75%) in mice relative to their weight at D0. This study was specifically approved by the Committee on the Ethics of Animal Experiments (CEEA) of Nord-Pas de Calais, France (permit number: CEEA#2016070717275082).

### Dual Energy X-Ray Absorptiometry (DEXA) Analysis

The fat mass, lean mass, and bone mineral content (BMC) of mice were analyzed using the Lunar PIXImus Mouse Densitometer (GE Healthcare, Madison, WI) according to a previously described protocol (27).

### Mineralized Bone Micro-CT Analysis

Tibiae extracted from CT and SBA mice were scanned using a Skyscan 1172 microCT device (Bruker MicroCT, Kontich, Belgium). The software suite provided by the manufacturer was used for image acquisition, reconstruction, analysis, and 3D visualisation (Skyscan 1172^©^, NRecon^©^, Dataviewer, CTAn^©^, CTVox™). Analysis of the tibia trabecular bone volume/tissue volume (BV/TV) ratio, cortical thickness (Cort.Th), trabecular thickness (Tb.Th), trabecular number (Tb.N) and trabecular spacing (Tb.Sp) of all animals were determined in a 0.5mm region beginning 250μm proximal to the proximal growth plate. More detailed description for the quantification of 3D microarchitecture of trabecular bone has been previously presented (28).

### Bone Marrow Adipose Tissue Content - Micro-CT Analysis

The BMAT and bone parameters analyzed by micro-CT of all mice were quantified using a previously published protocol (29) in the proximal tibia. To quantify the BMAT content, the same region of interest (ROI) was used as in the analysis of mineralized bone. After the first micro-CT scan, tibiae were decalcified in 4% formic acid/10% NBF (1:1), pH 7.4, for 48 h. Then bones were rinsed in distilled water and stained in the fume hood for 48 h in the aqueous staining solution (1% osmium tetroxide solution stabilized in a 2.5% dichromate potassium) at room temperature. Then, bones were rinsed for 48 h at room temperature in PBS with regular renewal of the solution. Osmium-stained bones were then stored at 4°C in PBS until the second micro-CT acquisition. The study focused on quantifying the percentage of the ratio of adipocyte volume to marrow volume (%, Ad.V/Ma.V). The data are presented in the form of the percentage of BMAT volume in marrow volume.

### Primary Bone Marrow Cell Cultures

Primary bone marrow cells were harvested from the tibiae and femurs of CT and SBA mice and grown for 7-10 days in αMEM media (PAN BIOTECH; P04-21050) with 15% fetal bovine serum (PAN BIOTECH; P30-3306), 1% penicillin/streptomycin (PAN BIOTECH; P04-85100) and 1% stable glutamine (PAN BIOTECH; P06-07100). After reaching full confluency, the plated BMSC population was differentiated into both osteoblasts and adipocytes according to the co-differentiation protocol described in a previously published article (30). The media containing co-differentiation inducers was replaced every two days during the 14 days of the co-differentiation experiment. This culture duration was chosen because of its brevity, which allowed for minimizing the time for cells to change after leaving the *in vivo* state.

### Raman Microspectroscopy

Cells were plated on calcium fluoride (CaF2) substrate, which is adapted to Raman analysis. The Raman microspectrometer was a LabRAM HR800 (Jobin-Yvon, France) instrument equipped with an XYZ motorized stage and a diode laser λ=785 nm. The Raman spectrometer was coupled with a microscope (BX40, Olympus). Raman acquisitions were performed with a water objective 100x lens (Nikon, numerical aperture = 1.1 Japan). The water immersion objective focused the laser on the cell within the micrometre scale on the centre of individual lipid droplets, where one spectrum corresponds to one adipocyte lipid droplet. A total of 1,252 lipid droplets were acquired over 109 adipocytes (**Table 1**), representing an average of 83.4 lipid droplets analyzed per well and an average of 7.2 adipocytes analyzed per well. Spectral acquisition was performed in the 400-1800 cm^-1^ range. The Raman signal was collected by a multichannel CCD detector (1024×256 pixels). The acquisition time was set at 60 s averaged 2 times per spectrum (total acquisition time = 120 sec). Raman spectra were processed using Labspec software (HORIBA, Jobin-Yvon, France). A Savitzky–Golay smoothing filter was applied to all Raman spectra. The band areas were integrated over defined Raman shift regions in the spectrum using a sum filter. The filter calculates the areas within the chosen borders and the background is subtracted by taking the baseline from the first to the second border. The band area was estimated at 1441 cm^-1^ (peak-ROI 1400-1500 cm^-1^) and 1654 cm^-1^ (peak-ROI 1620-1694 cm^-1^). The Raman bands at 1441 cm^-1^ and 1654 cm^-1^ are assigned to vibrations CH2 and C=C, respectively (31). The unsaturation ratio was calculated as the ratio of band areas 1654 cm^-1^/1441 cm^-1^ (31).

**Table 1:** Details of lipid droplets studied in each condition of the 10-week and 4-week protocols.

Condition	Number of lipid droplets = number spectra over 3 wells	Number of adipocytes analysed over 3 wells per condition
10-week		
CT	519	41
SBA24%	111	13
SBA18%	299	30
SBA12%	134	10
SBA0%	189	15
Total	1252	109
4-week		
CT	45	18
SBA18%	109	25
Total	154	43

### Mineralization Quantification

The sample mineralization level was quantified using the same protocol previously described (30). Briefly, cells were harvested in a solution of PBS 1X/Triton 0.2%/HCl 6 M and disrupted by sonication. After, 5 μl of HCl 6 M was added to 95 μl of each sample and incubated overnight at 4°C. Following by centrifugation at 1500 × g for 5 min, the protein content was determined using the DC protein assay kit BioRad. The mineralization content was quantified.

### RNA Extraction, Reverse Transcription and Real-Time PCR

Total RNA was extracted from BMSC cultures by the previously described protocol (30). Reverse transcription and RT–PCR were performed as previously described (32). The sequences of the primers (TibMolBiol, Berlin, Germany), annealing temperature, product length, and GenBank reference for each of the analyzed genes are shown in **Table 2**.

**Table 2:** Primer sequences and conditions of quantitative RT–PCR.

Gene	Primer Sequences	Annealing temperature	Product length	GenBank
*18S*	F: ATTCCGATAACGAACGAGAC R: GCTTATGACCCGCACTTACT	60°C	297 bp	X03205
*Gapdh*	F: GGCATTGCTCTCAATGACAA R: TGTGAGGGAGATGCTCAGTG	62°C	200 bp	NM_008084
*Pparg2*	F: TCGCTGATGCACTGCCTATG R: GAGAGGTCCACAGAGCTGATT	60°C	103 bp	NM_011146
*Adipoq*	F: TGTTCCTCTTAATCCTGCCCA R: CCAACCTGCACAAGTTCCCTT	60°C	104 bp	NM_009605
*Lep*	F: GAGACCCCTGTGTCGGTTC R: CTGCGTGTGTGAAATGTCATTG	60°C	139 bp	NM_008493
*Plin1*	F: CACCATCTCTACCCGCCTTC R: GGGTGTTGGCGGCATATTC	58°C	149 bp	NM_001113471.1
*Glut4*	F: ACTCTTGCCACACAGGCTCT R: AATGGAGACTGATGCGCTCT	62°C	174 bp	NM_009204
*Runx2*	F: GCCGGGAATGATGAGAACTA R: GGACCGTCCACTGTCACTTT	62°C	200 bp	NM_001146038.2
*Bglap*	F: AAGCAGGAGGGCAATAAGGT R: CGTTTGTAGGCGGTCTTCA	60°C	364 bp	L24431
*Sp7*	F: ATGGCGTCCTCTCTGCTTG R: TGAAAGGTCAGCGTATGGCTT	54°C	237 bp	NM_130458
*Col1a1*	F: GGTGAGCCTGGTCAAACGG R: ACTGTGTCCTTTCACGCCTTT	60°C	189 bp	NM_007743
*Sirt1*	F: TAGGGAACCTTTGCCTCATC R: GGCATATTCACCACCTAGCC	51°C	100 bp	NM_019812.2

### Statistical Analysis

The 10-week study was performed with 6 samples per condition, except for the mineralization experiment, which had 4 replicated per group. The 4-week study was performed with 5 samples per condition. The median and interquartile range were calculated for the groups. Due to the low number of replicates, the normality of the data could not be tested. In 10-week study, we performed Kruskal-Wallis One-Way ANOVA test to show the significance between all the conditions. Additionally, we conducted Dunn’s test in order to see the relationships between specific pairs of conditions. For the 4-week studies we performed Mann–Whitney’s test to see the statistically significant relationship between the two particular conditions. All tests were performed using GraphPad Prism software. Differences with p<0.05 were considered statistically significant.

## Results

### The 10-Week SBA Protocols Induced a Significant Decrease in Mouse Body Weight (12%, 18%, 24%) and Were Shown to Cause Changes Not Only in Fat Mass But Also in Lean Mass and Bone Mineral Composition

**Figure 1:**
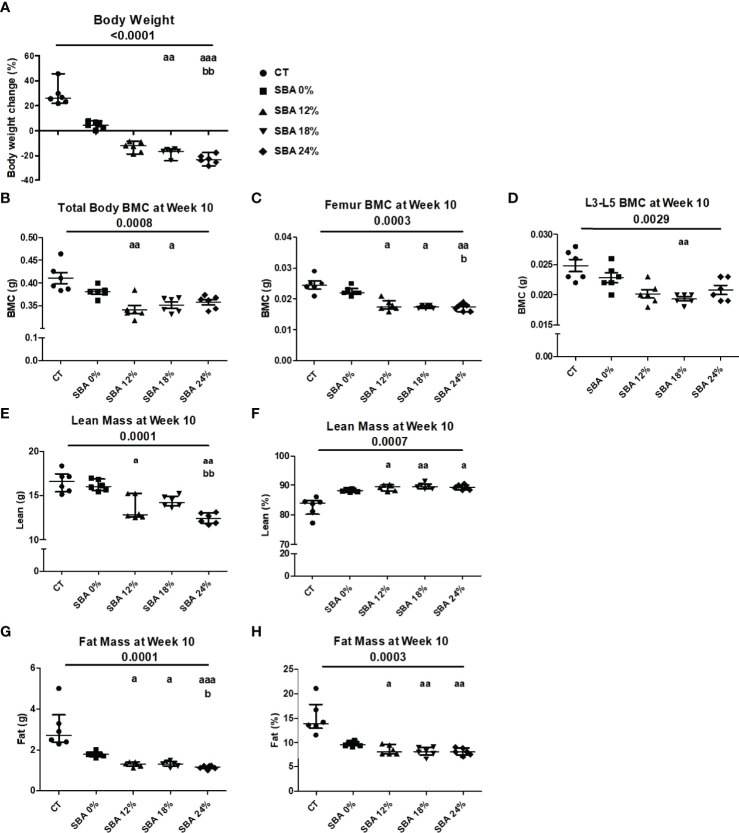
Body mass, BMC, fat and lean mass in mice after 10 weeks of SBA protocols of different severity. The body weight and composition analysis were performed on standard condition (CT), constant weight group (SBA 0%) and three weight loss groups with different severity levels (SBA 12%, 18% and 24%). **(A)** Graph represents a percentage body weight loss on mice between day 0 and 10 weeks of SBA protocol; **(B-D)** Study of total mouse body mineral content (BMC), femur BMC and vertebra (L3-L5) BMC, respectively, were evaluated for each animal and each condition after 10 weeks of SBA protocol.; **(E, F)** Lean mass content presented in grams and % of total mass, respectively was evaluated for each mouse and condition after 4 weeks of SBA protocol; **(G, H)** Fat mass content presented in grams and % of total mass, respectively was evaluated for each mouse and condition after 4 weeks of SBA protocol. Data represent median and interquartile range; n = 6. Statistical analysis was performed using Kruskal-Wallis One-Way ANOVA test and Dunn’s test; a - p < 0.05, aa - p < 0.005 and aaa - p < 0.0005 when compared to the CT group (b – when compared to the SBA 0% group).

To study the effect of energy deficit severity on bone and BMSCs, we performed four different SBA protocols on female mice. We induced 0%, 12%, 18%, and 24% weight loss from the initial mouse weight over 10 weeks. Prior to the study, mice were randomized into five experimental groups that did not display significant differences in body weight at Day 0. Mice from the control group (*ad libitum* and not separated) displayed a significant body weight increase (+22.42% vs. D0) after 10 weeks (**Figure 1A**). In the SBA 0% group, no significant weight change (+4.39% vs. D0) was observed, as this condition did not imply weight change (**Figure 1A**). In all other SBA groups, we observed a significant decrease in weight after 2 weeks of protocol and this decrease was maintained until the end of the 10 weeks (SBA 12% (-12.97%) vs. D0; SBA 18% (-17.27%) vs. D0; and SBA 24% (-23.17%) vs. D0) (p<0.005 vs. D0). The results of DEXA analysis showed a significant decrease in total body bone mineral content (BMC) only in light (12%) and moderate (18%) weight loss conditions (SBA 12% -17% vs. CT; SBA 18% -15% vs. CT) (**Figure 1B**). The study of the BMC at specific sites, revealed that all three weigh loss conditions induced a significant decrease in femur BMC - SBA 12% (-27% vs. CT), 18% (-29% vs. CT) and 24% (-30% vs. CT) (**Figure 1C**). However, only the SBA 18% group showed a significant decrease in the BMC of lumbar vertebrae (L3-L5) (SBA 18% -23% vs. CT) (**Figure 1D**). The BMC decrease was potentially associated with a decrease in lean absolute mass in mice from the SBA groups 12% (-19% vs. CT) and 24% (-22% vs. CT). This parameter was significantly reduced only in weight loss conditions, while in the SBA 18% group, lean absolute mass just showed a tendency to decrease without statistically significant change (**Figure 1E**). However, study of lean percentage (%) of total mouse mass, showed a significant increase in all three weigh loss conditions (SBA 12%. 18% and 24%). This was due to a significant decrease in fat mass in same conditions (on average -53% vs. CT). Fat absolute mass in weight loss conditions exhibited a great decrease (59-64%, p 0.0001 vs. CT) (**Figure 1F**). These data highlight that absolute lean mass and fat mass parameters can be associated with BMD under weight loss conditions, as a significant decrease in whole-body BMC or femoral/vertebral BMC together with lean and fat mass was observed only in the weight loss mice.

### The 18% and 24% SBA Protocols Resulted in a Significant Decrease in Trabecular and Cortical Thickness, Without Any Increase in Bone Marrow Adiposity

**Figure 2:**
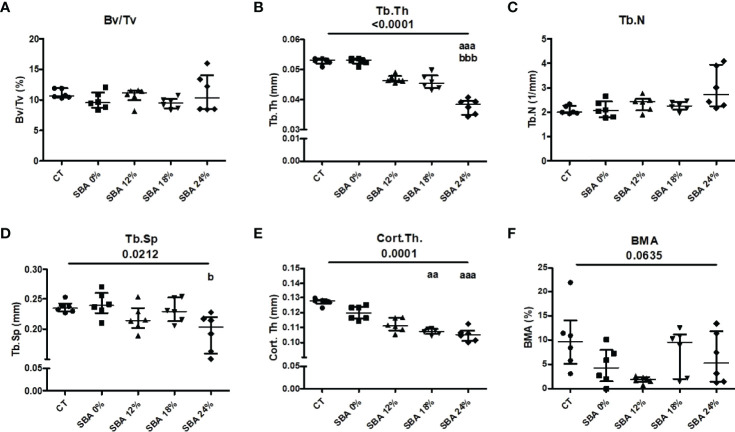
The results of micro-CT analysis of tibia, showing different bone parameters after exposure to 10 weeks of SBA protocols at different levels of severity. **(A)** Bone volume fraction (BV/TV) was expressed as a percentage on mineralized tissue in each condition after 10 weeks; **(B–E)** Trabecular thickness (Tb.Th), trabecular number (Tb.N) and trabecular spacing (Tb.Sp), and cortical thickness (Cort.Th), respectively, were evaluated for each animal and each condition after 10 weeks of SBA protocol (expressed in millimetres); **(F)** Bone marrow adiposity (BMA) was calculated as a percentage of BMAT volume in marrow volume. Data represent median and interquartile range; n = 6. Statistical analysis was performed using Kruskal-Wallis One-Way ANOVA test and Dunn’s test; a - p < 0.05, aa - p < 0.005 and aaa - p < 0.0005 when compared to the CT group (b – when compared to the SBA 0% group).

To determine the effect of SBA severity on bone microarchitecture, we conducted proximal tibia micro-CT analysis. Bone volume fraction (BV/TV) showing the relative volume of mineralized trabecular bone was not affected by the SBA protocols (**Figure 2A**). Trabecular thickness (Tb.Th) was significantly decreased only under severe weight loss conditions (SBA 24%: -29% vs. CT) (**Figure 2B**). In contrast with the thickness of the trabecular bone, the trabecular number (Tb.N) and trabecular spacing (Tb.Sp) were not significantly affected, even for the 24% group (**Figures 2C, D**). One of the bone fragility parameters was assessed through the study of cortical thickness (Cort.Th), which is affected by the SBA protocol. Indeed, the SBA 18% and 24% caused 16% and 17% decreases in this parameter, respectively (p 0.0001 vs. CT) (**Figure 2E**), demonstrating the potential link between moderate and severe energy deficit and decrease in Cort.Th. The results of bone marrow adiposity (BMA) showed a high heterogeneity of the samples in each group. Only the SBA 12% group exhibited a tendency of BMA decrease by 82% (p<0.1, **Figure 2F**).

### SBA Protocol Resulted in Rapid Adipogenesis of BMSCs and Weight Loss Conditions Were Associated With a Decrease in Lipid Unsaturation Levels

**Figure 3:**
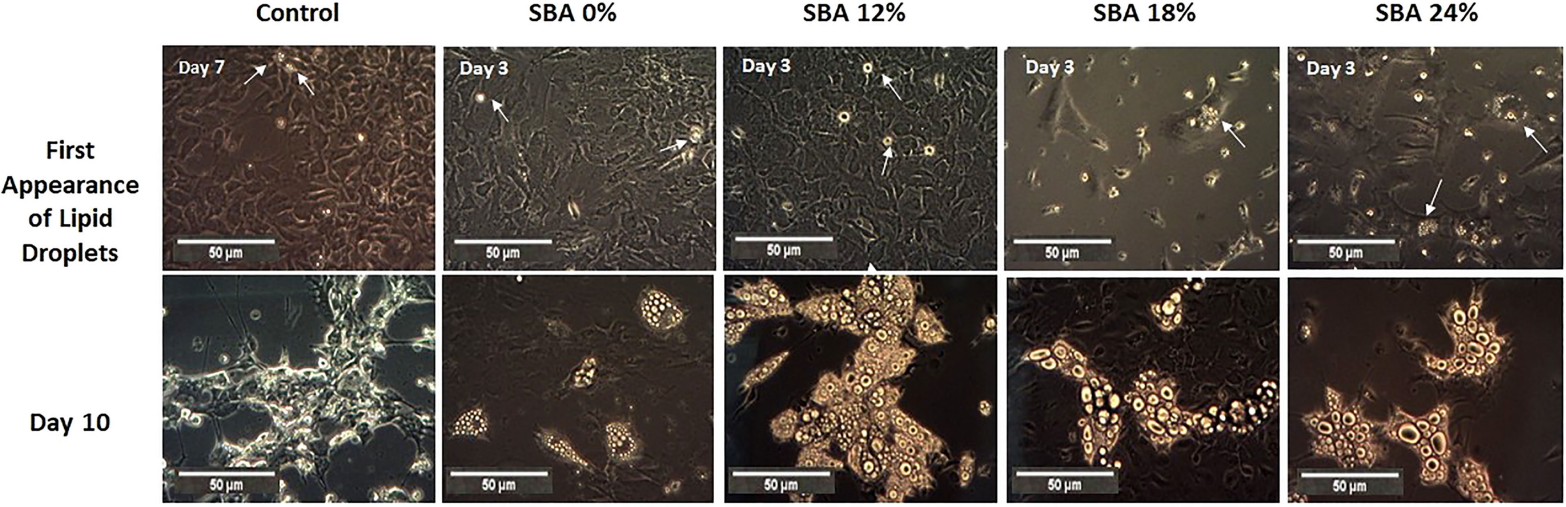
Overview of adipocyte first appearance and size. A. Light microscope images of cells from each condition were taken on different points of differentiation showing the first appearance of lipid droplets and adipocytes at day 10.

Throughout the co-differentiation experiment, cells extracted from SBA and control mice were observed under light microscopy. BMSCs extracted from CT mice started to form lipid droplets after the 7^th^ day of co-differentiation. Cells extracted from SBA mice (0%, 12%, 18%, and 24%) showed the first appearance of lipid droplets much faster, after the 3^rd^ day of co-differentiation (**Figure 3**). This finding suggests that the SBA protocol induces *in vivo* changes in BMSC commitment and that these cells are potentially committed to differentiating into adipocytes. After qualitative study (microscopic observations) of adipocytes on Day 10 of differentiation, BMSCs extracted from the SBA 12%, 18%, and 24% groups globally seemed to generate more numerous and larger adipocytes compared to the CT or SBA 0% groups (**Figure 3**). Additionally, Raman spectroscopy analysis was performed to characterize more of the adipocytes from different groups. The lipid unsaturation levels in the SBA 12%, 18%, and 24% groups were significantly lower than those in the SBA 0% group, showing that the severity of energy deficit can lead to a decrease in lipid unsaturation levels (**Supplementary Figure 1**).

### Only SBA 18% and 24% Protocols Resulted in an Increase in Adipogenesis and Subsequent Decrease in Osteoblastogenesis

**Figure 4:**
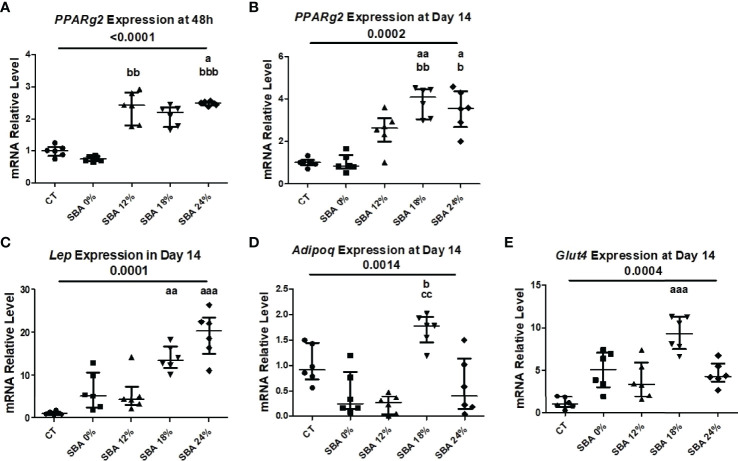
10-week SBA protocol induced an increase in adipogenesis. **(A, B)** Relative mRNA expression level of early adipogenic marker gene (*Pparg2*) in CT and SBA groups (0%, 12%, 18%, 24%), cells were cultured for 48 hours in standard growth media and for 14 days were exposed to co-differentiation media, respectively; **(C-E)** Relative mRNA expression level of other adipogenic marker genes (*Lep, Adipoq*, and *Glut4*, respectively) in CT and SBA groups (0%, 12%, 18%, 24%) after exposure to co-differentiation media during 14 days. Data represent median and interquartile range; n=6. Statistical analysis was performed using Kruskal-Wallis One-Way ANOVA test and Dunn’s test; a - p < 0.05, aa - p < 0.005 and aaa - p < 0.0005 when compared to the CT group (b – when compared to the SBA 0% group; c – when compared to SBA 12% group). Peroxisome proliferator-activated receptor gamma 2 (*Pparg2*), leptin (*Lep*), adiponectin (*Adipoq*), facilitated glucose transporter member 4 (*Glut4*).

To confirm the commitment of BMSCs to the adipocyte lineage, the mRNA levels of adipocytic markers in the SBA and CT groups were analyzed. The marker gene relative mRNA level was measured 48 hours after plating (adherent cells) and at day 14 of co-differentiation. This culture duration was chosen because of its brevity, which minimized the time for cells to change after leaving the *in vivo* state. Interestingly, after 48 hours of adhesion in a standard medium, the unstimulated BMSCs from mice of SBA 24% protocols already exhibited high levels of peroxisome proliferator-activated receptor gamma 2 (*Pparg2*) mRNA (+125% vs CT) (**Figure 4A**), showing a potential commitment to adipogenic differentiation prior to co-differentiation stimulation and thus probably *in vivo*. The SBA 0% group showed similar expression level of *Pparg2* to CT and SBA 12% and 24% conditions, also showed significant increase in *Pparg2* relative mRNA level vs. SBA 0%. Furthermore, *Pparg2* mRNA levels were similarly enhanced after 14 days of co-differentiation in severe weight loss group (SBA 24%: +236% vs. CT) (**Figure 4B**). Additionally, the SBA 18% group demonstrated the highest upregulation of *Pparg2* (+ 285%vs. CT and vs. SBA 0%) (**Figure 4B**). Other marker genes characterizing cell commitment to adipogenic differentiation (leptin (*Lep)* and facilitated glucose transporter member 4 (*Glut4)*) exhibited an increase in the moderate and severe weight loss groups (SBA 18% - *Lep* +1292% vs CT, *Glut4* +807% vs. CT; SBA 24% - *Lep* +1864% vs. CT) (**Figures 4C, E**). The adiponectin (*Adipoq)* mRNA level was found to be upregulated only in the SBA 18% group (+53% vs. SBA 0%) (**Figure 4D**). However, the 12% weight loss condition displayed the lowest mRNA level of *Adipoq* (**Figure 4D**).

**Figure 5:**
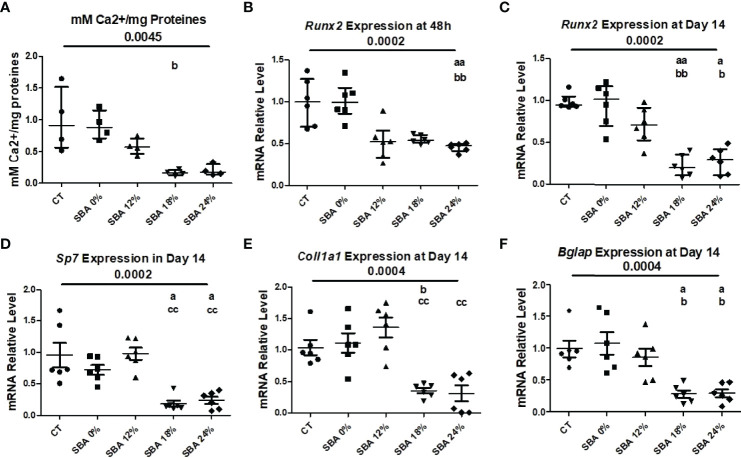
10-week SBA protocol induced a decrease in osteoblastogenesis. **(A)** Mineralization level of CT and SBA groups (0%, 12%, 18%, 24%) quantified by measuring the calcium to protein ratio; **(B, C)** Relative mRNA expression level of early osteoblastogenic marker gene (*Runx2*) in CT and SBA groups (0%, 12%, 18%, 24%), cells were cultured for 48 hours in standard growth media and for 14 days were exposed to co-differentiation media, respectively; **(D-F)** Relative mRNA expression level of other osteoblastogenic marker genes (*Sp7, Col1a1*, and *Bglap*, respectively) in CT and SBA groups (0%, 12%, 18%, 24%) after exposure to co-differentiation media for 14 days. Data represent median and interquartile range; Mineralisation study n = 4, qPCR n = 6. Statistical analysis was performed using Kruskal-Wallis One-Way ANOVA test and Dunn’s test; a - p < 0.05, aa - p < 0.005 when compared to the CT group (b – when compared to the SBA 0% group; c – when compared to SBA 12% group). Runt-related transcription factor 2 (*Runx2*), osterix (*Sp7*), collagen1a1 (*Col1a1*), osteocalcin (*Bglap*). *Sirt1 is sustainably decreased in BMSCs from SBA mice (18% and 24%)*.

In our study, we wanted to determine whether the severity of SBA causes an imbalance in BMSC lineage commitment and whether upregulation of adipogenesis is achieved at the expense of osteoblastogenesis. Thus, after 14 days of co-differentiation, the mineralization level of CT and SBA cells was studied by quantifying the calcium to protein ratio. The results demonstrated a strong alteration of mineralization capabilities in moderate and severe weight loss SBA conditions (18% and 24%, on average -82% vs. CT) (**Figure 5A**). However, this trend was not significant. Only 18% weigh loss condition exhibited significant decrease in mineralization vs. SBA 0%. The mRNA level analysis showed that the early marker of osteoblastogenesis [runt-related transcription factor 2 (*Runx2)*] was significantly reduced in severe weight loss condition (SBA 24% -55% vs. CT) 48 hours after cell plating in standard media and after 14 days in co-differentiation medium both moderate and severe conditions induced changes in *Runx2* mRNA level (SBA 18% -75% vs. CT; SBA 24% -70% vs. CT) (**Figures 5B, C**). Interestingly, further study of other markers of osteoblastogenesis [collagen1A1 (*Col1a1*), osteocalcin (*Bglap*) and osterix (*Sp7*)] at the end of co-differentiation revealed a reduction in mRNA levels only in the moderate and severe weight loss groups (*Col1a1* - SBA 18% -77% vs SBA 0%, SBA 24% -83% vs. SBA 12%; *Bglap* - SBA 18% -73%, SBA 24% -72% vs. CT; *Sp7* - SBA 18% -81%, SBA 24% -77% vs. CT) (**Figures 5D–F**). Moreover, the results suggest that an increase in adipogenesis occurs at the expense of osteoblastogenesis and that BMSCs extracted from SBA mice are apparently more committed to adipogenic lineage.

**Figure 6:**
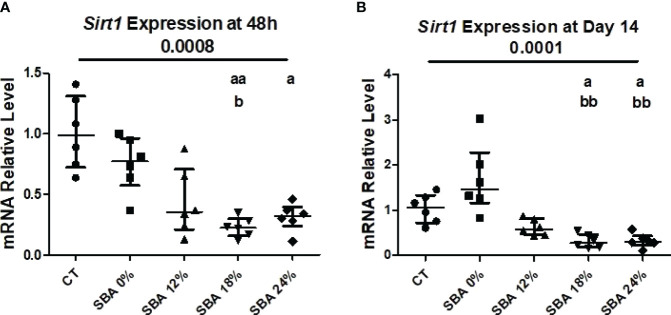
10-week SBA protocol induced a suppression of SIRT1 mRNA level. **(A, B)** Relative mRNA expression level of sirtuin type 1 (*Sirt1*) in CT and SBA groups (0%, 12%, 18%, 24%), cells were cultured for 48 hours in standard growth media and for 14 days were exposed to co-differentiation media, respectively. Data represent median and interquartile range; n = 6. Statistical analysis was performed using Kruskal-Wallis One-Way ANOVA test and Dunn’s test; a - p < 0.05, aa - p < 0.005 when compared to the CT group (b – when compared to the SBA 0% group).

Recently, we demonstrated that the 18% SBA protocol favors BMSC lineage commitment towards adipogenesis and a decrease in *Sirt1* level and activity, a potential regulator of BMSC differentiation (26). In the present study, we examined the impact of weight loss severity on the expression level of *Sirt1* mRNA. The results of the SBA 18% protocol showed a 67% decrease (vs. CT) in *Sirt1* mRNA in BMSCs after 48 hours of adhesion in growth media and a 75% decrease (vs. CT) after 14 days of co-differentiation (**Figure 6**). This finding confirmed our previously published data. Additionally, the *Sirt1* mRNA level was significantly altered in the 24% groups (-60% vs. CT) after 48 hours of adhesion and after 14 days of co-differentiation (-68% vs. CT) (**Figure 6**). So, decrease in *Sirt1* mRNA levels under moderate and severe weight loss conditions could be associated with an increase in adipogenic mRNA markers and a subsequent downregulation of osteoblastogenesis in BMSCs.

### The 4-Week SBA Protocol Induced a Significant Decrease in Mouse Body Weight (18%) and Was Shown to Cause Changes Only in Fat Mass and Trabecular Thickness

Long-term SBA protocols (10-week duration) have been shown to induce changes in mouse BMSC differentiation capacity; however, we were interested in the duration of energy deficit affecting the mouse bone composition and BMSC differentiation capacity. Therefore, we conducted a 4-week SBA protocol. In the 10-week study detailed above, the SBA 18% group exhibited alterations in bone parameters and in BMSC differentiation capacity, confirming the hypothesis that adipogenesis is upregulated in these cells at the expense of osteoblastogenesis. Based on these findings, we compared CT mice with 18% weight loss mice after exposure to the SBA protocol for 4 weeks.

**Figure 7:**
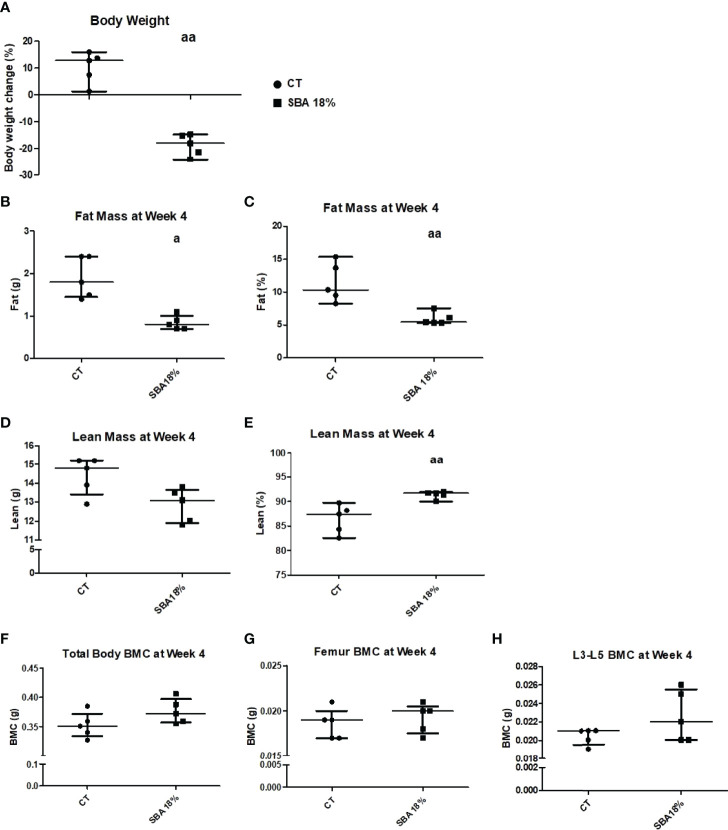
Body mass, BMC, fat and lean mass in mice after 4 weeks of SBA protocol. The body weight and composition analysis were performed on standard condition (CT) and weight loss group (SBA 18%). **(A)** Graph represents a percentage body weight loss on mice between day 0 and 4 weeks of SBA protocol; **(B, C)** Fat mass content presented in grams and % of total mass, respectively was evaluated for each mouse and condition after 4 weeks of SBA protocol; **(D, E)** Lean mass content presented in grams and % of total mass, respectively was evaluated for each mouse and condition after 4 weeks of SBA protocol; **(F-H)** Study of total mouse body mineral content (BMC), femur BMC and vertebra (L3-L5) BMC, respectively, evaluated for each animal and each condition after 4 weeks of SBA protocol. Data represent median and interquartile range; n = 5. Statistical analysis was performed using Mann–Whitney’s test; a - p < 0.05, aa - p < 0.005, when compared to the CT group.

The mouse body weight showed a significant decrease in percentage body weight loss in the SBA 18% group vs. CT (**Figure 7A**). After the study of body composition, it was concluded that the 4-week SBA protocol induced alterations in mouse fat absolute mass (-50% vs. CT), with no change in BMC or lean absolute mass (**Figure 7**). However, the significant decrease in fat % of total mouse weight was accompanied with significant increase in lean % mass (**Figures 7C, E**).

**Figure 8:**
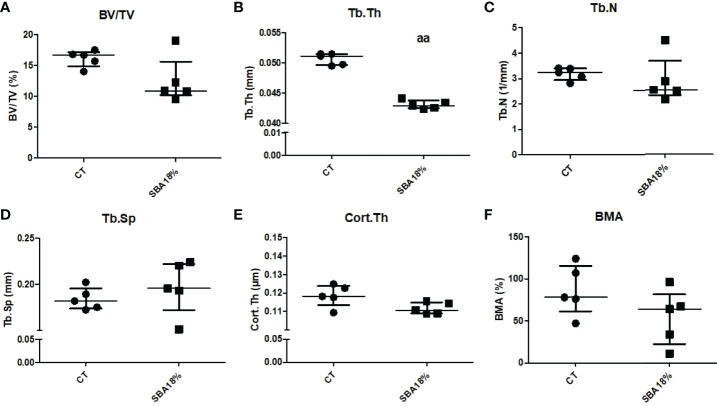
The results of micro-CT analysis of tibia, showing different bone parameters after exposure to 4 weeks of SBA protocol. **(A)** Bone volume fraction (BV/TV) was expressed as a percentage on mineralized tissue in each condition after 4 weeks; **(B-E)** Trabecular thickness (Tb.Th), trabecular number (Tb.N) and trabecular spacing (Tb.Sp), and cortical thickness (Cort.Th), respectively, were evaluated for each animal and each condition after 4 weeks of SBA protocol (expressed in millimetres); **(F)** Bone marrow adiposity (BMA) was calculated as a percentage of BMAT in marrow volume. Data represent median and interquartile range; n = 5. Statistical analysis was performed using Mann–Whitney’s test. In C, aa - p < 0.005 when compared to CT group.

Furthermore, the bone microarchitecture results showed that the short SBA protocol induced changes in Tb.Th (-16% vs. CT) (**Figure 7B**) to a similar extent as the 10-week 18% weight loss protocol (**Figure 2B**). The 4-week SBA protocol also had no effect on BV/TV, Tb.N and Tb.Sp (**Figures 8A, B, D**), similar to SBA 18% group in 10-week protocol (**Figure 2**). However, in contrast with 10-week protocol, short duration of SBA had no effect on Cort.Th (**Figure 8E**). Additionally, bone marrow adiposity was not affected by the 4-week SBA protocol (**Figure 8F**). Thus, the 4-week protocol was not enough to induce changes in cortical bone microarchitecture, unlike the 10-week protocol.

### Reduced Length of SBA Protocol Did Not Result in Alterations of BMSC Differentiation Capacity or Changes in Sirt1 Expression

**Figure 9:**
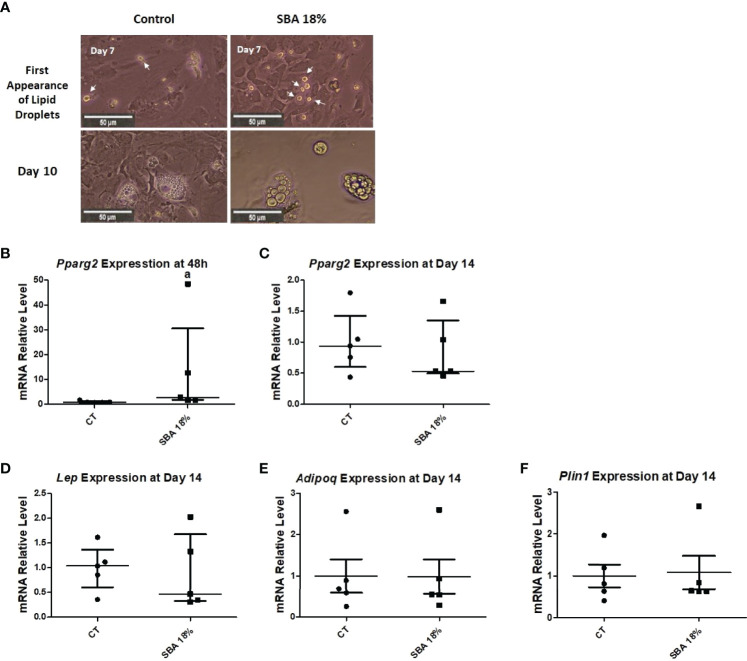
Study of adipocyte first appearance, size and adipogenic gene expression in BMSCs. **(A)** Light microscopic images of cells from each condition were taken at different time points of co-differentiation showing the first appearance of lipid droplets (day 7) and adipocytes at Day 10; **(B, C)** Relative mRNA expression level of early adipogenic marker gene (*Pparg2*) in CT and SBA (18%) groups, cells were cultured for 48 hours in standard growth media and for 14 days were exposed to co-differentiation media, respectively; **(D–F)** Relative mRNA expression level of other adipogenic marker genes (leptin, adiponectin, and perilipin, respectively) in CT and SBA groups (18%) after exposure to co-differentiation media for 14 days. Data represents median and interquartile range; n = 5. Statistical analysis was performed using Mann–Whitney’s test. In **(B)**, a - p < 0.05 when compared to CT group. Peroxisome proliferator-activated receptor gamma 2 (*Pparg2*), leptin (*Lep*), adiponectin (*Adipoq*), perilipin (*Plin1*).

After BMSCs were extracted from bone marrow and co-differentiation was induced, the cells were studied under a light microscope. Images showed that the first appearance of lipid droplets took place on the 7^th^ day of co-differentiation in both the CT and SBA 18% groups (**Figure 9A**). Additionally, after 10 days of differentiation, there was no difference observed in the accumulation of adipocytes in SBA 18% vs. CT (**Figure 9A**). Therefore, the 4-week SBA protocol did not induce a shift of BMSCs towards adipocyte differentiation, as suggested by the results of the 10-week experiments. Similarly, it did not induce changes in lipid accumulation capacities. Raman spectroscopy showed no difference in lipid unsaturation levels between the control and SBA 18% groups (**Supplementary Figure 2**). Similar to the 10-week SBA study, we analyzed the relative mRNA levels of adipogenic/osteoblastogenic genes in extracted BMSCs. The results of the early adipogenic marker gene (*Pparg2*) showed that after 48 hours of proliferation in standard growth media, BMSCs extracted from SBA 18% mice had significantly increased expression levels (+350% vs. CT) (**Figure 9B**). However, this upregulation of *Pparg2* expression was not long term and after 14 days of co-differentiation, there was no significant difference observed between the two conditions (**Figure 9C**). The analysis of late adipogenic marker genes (*Lep*, *Adipoq*, *Plin1*) demonstrated no change in the SBA 18% group compared to the CT group after 14 days of co-differentiation (**Figures 9D–F**).

**Figure 10:**
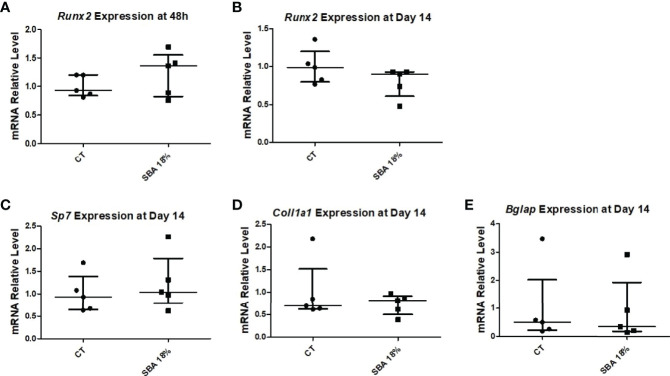
4-week SBA protocol did not induce changes in osteoblastogenesis. **(A, B)** Relative mRNA expression level of early osteoblastogenic marker gene (RUNX2) in CT and SBA groups (18%), respectively, cells were cultured for 48 hours in standard growth media and for 14 days were exposed to co-differentiation media, respectively; **(C-E)** Relative mRNA expression level of other osteoblastogenic marker genes (osterix, collagen1, and osteocalcin, respectively) in CT and SBA (18%) groups after exposure to co-differentiation media for 14 days. Data represent median and interquartile range; n = 5. Statistical analysis was performed using Mann–Whitney’s test. Runt-related transcription factor 2 (*Runx2*), osterix (*Sp7*), collagen1a1 (*Col1a1*), osteocalcin (*Bglap*).

The next step was to determine the expression level of osteoblastic marker genes in the CT and SBA 18% groups. The early osteoblastogenic marker (*Runx2*) was not affected by 4 weeks of CR after 48 hours of incubation in proliferation media or after 14 days of exposure to co-differentiation media (**Figures 10A, B**). Moreover, other osteoblastogenic marker genes (*Col1a1*, *Sp7* and *Bglap*) were also not altered by the short-term SBA protocol (**Figures 10C–E**). The results of this study suggest that the 4-week protocol does not induce alterations in BMSC differentiation capacity.

**Figure 11:**
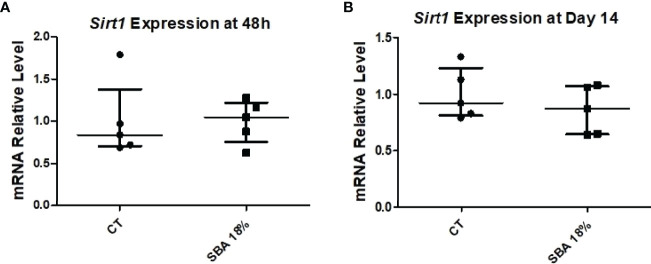
4-week SBA protocol induced no change in *Sirt1* expression. **(A, B)** Relative mRNA expression level of sirtuin type 1 (*Sirt1*) in CT and SBA groups (18%), cells were cultured for 48 hours in standard growth media and for 14 days were exposed to co-differentiation media, respectively. Data represent median and interquartile range; n = 5. Statistical analysis was performed using Mann–Whitney’s test.

To confirm the association between *Sirt1* expression and BMSC lineage commitment, we examined relative mRNA levels in the 4-week SBA 18% group vs. the CT group. The findings showed that the expression of *Sirt1* was not affected by the short SBA protocol, further suggesting its involvement in BMSC fate decisions (**Figure 11**).

## Discussion

### Ten Weeks of 18% and 24% Body Weight Loss Induce an Alteration of Bone Mineral Content Without Increase in Bone Marrow Adiposity

We recently demonstrated that a long-term energy deficit characterized by a moderate weight loss (18%) in mice is associated with bone loss and an increase in BMA (26). In this study, we sought to determine the impact of a different degree of severity on bone architecture, BMA, and then to identify the potential molecular mechanisms involved in BMA regulation in this context. The mice were exposed for 10 weeks to a separation-induced increase in energy expenditure and a time-restricted feeding that prevented compensatory feeding and resulted in significant weight loss (SBA) (27). Results of mice body parameters showed similar fat mass decrease in all SBA groups compared to the control group. The 12%, 18%, and 24% groups displayed a decrease in BMC at least in femur. These results suggest that a significant weight loss over a long period of time (10 weeks) is required to induce changes in mineralized bone (**Figure 1**). Moreover, these bone changes are not directly related to fat mass because they are not shown in the 0% group. This was already shown in adult male mice by Mitchell et al. (33). They demonstrated that different levels of CR 10%, 20%, 30%, and 40% in male mice induced body weight losses of around 0%, 11%, 22%, and 28%, respectively after 12 weeks (33). These weight changes were associated to fat mass decrease of approximately 12% for 10% CR and 30 to 35% for the other groups. In this study, no significant changes in BMC, trabecular bone microarchitecture, and cortical bone were detected. Unfortunately, BMA was not determined. The conflicting results on bone in the present study and that of Mitchell could be due to sex, age, and protocol differences. Furthermore, Mitchell’s study also showed the lack of relationship between fat mass decrease and bone in energy deficit experiment.

Previously, we showed that the SBA protocol of 18% weight loss results in an increase in BMA (26). Interestingly, in the current study, BMA appeared to be altered only in one weight loss condition. BMA displayed a decrease (not significant) in SBA mice with a light weight loss (12%) (**Figure 2F**). Firstly, the apparent discrepancy between the two studies could be explained by the fact that we observed a high heterogeneity in BMA among mice of the same group. Secondly, in the first study, BMA was assessed using histological (2D) approaches with slices focused on a specific area of the proximal tibia and with a specific orientation, while this study used 3D assessment on a larger volume including the area analyzed in the previous study. So, the increase observed in a specific area and the lack of significant changes observed on the large volume suggest that very localized changes could occur in the density of adipocytes in the marrow. Various studies demonstrated an increase in BMA associated with bone alteration in anorexic patients (9, 10, 13). In rodent, models of food or calorie restriction demonstrated that 9 weeks of a 30% restriction on food in 3-week-old male mice induced low BV/TV (-11%), Cort.Th (-27%), and body weight (-40%), which were accompanied with high BMA (700%) compared to CT mice (34). Also, it has been shown that 6 weeks of a 30% calorie-restricted diet in 9-week-old female mice induced an increase in tibia BMA (700%) compared to that in the *ad libitum* group (35). Interestingly, in these two studies, the high increase in BMA was associated with a body weight increase of 80% vs. day 0 (34) or a final body weight similar to that of day 0 of the experiment (35). The difference in our BMA results compared to these two studies (34, 35) may be explained by the fact that we used young adult female mice, while the first one investigated growing male mice (34) and by the difference in body weight loss relative to that on day 0 (33, 34). In addition, it is reported that an extensive CR in rabbits can cause a loss of BMAT (34). A similar finding has been observed with severe anorexic patients (36). Thus, the sex of animals and level of body weight loss may strongly affect BMA. In contrast, a calorie restriction protocol (low carbohydrate feeding) for 12 weeks on 6-month-old Sprague–Dawley female rats, demonstrated that restricted rats displayed body weight loss (20% vs. day 0 and 25% vs. CT rats), a low volumetric BMD at the proximal tibia (-14%) and only a two-fold increase in BMA measured at the distal femur (37). More recently, male Sprague–Dawley rats (8 months old) exposed to 40% calorie restriction during 12 months, displayed 40% body weight loss vs. CT rats, which resulted in a decrease in the bone mineral index (30% vs. CT rats) and an increase in BMA at the proximal tibia (20% vs. CT rats) (38). Conversely, 10 weeks of CR in 14-week-old male mice led to a body weight loss of 30% (relative to CT mice) and a total disappearance of bone marrow adipocytes in the distal femur (34). Altogether, these data suggest that restriction-induced changes in BMA and bone quality may be modulated by severity of protocol, body weight loss, age, sex, and duration of restriction.

### Ten Weeks of 18% and 24% Body Weight Loss Induce an Imbalance Between Osteoblastogenesis and Adipogenesis

Due to the fact that, in the current study, bone alterations were not associated to a high BMA, we hypothesised that the commitment of BMSCs towards the adipogenic pathway could impact bone mass even if adipocytes are not able to reach a mature state. Therefore, it was important to study BMSC differentiation to obtain a more precise understanding of the cell commitment towards the adipogenic or osteoblastogenic pathway.

Given that osteoblasts and adipocytes are derived from a common mesenchymal progenitor, skeletal stem cells (39, 40), we tested the hypothesis that the degree of severity of body weight loss could impact the differentiation capacity of BMSCs extracted from SBA mice. After just 48 hours of adhesion in a standard proliferative medium, the unstimulated BMSCs from SBA mice with body weight loss (12%, 18%, and 24%) exhibited high levels of *Pparg2* mRNA even if not significantly for the 18% group (**Figure 4A**). This finding suggests that extracted BMSCs display commitment to the adipocytic lineage. As shown in **Figures 3** and **4**, SBA weight loss protocols enhance BMSC adipogenesis *in vitro*, especially for SBA 18% and 24% groups. The mRNA level of adiponectin was dramatically decreased in SBA mice with light weight loss (12%) and this finding could be associated with a significant decrease in BMA under the same weight loss conditions. Significant loss of mature adipocytes *in vivo* could potentially affect the level of adiponectin production and *Adipoq* mRNA levels (41). Raman spectroscopy analysis (**Supplementary Figure 1**) further revealed that SBA protocol results in a decrease of unsaturated lipids ratio in BMSC-derived adipocytes. This suggests that energy deficit induces a long-lasting alteration of lipid quality regardless the level of body weight loss. Our analysis of osteoblastogenesis in BMSCs (**Figure 5**) suggests that only 18% and 24% weight loss groups induce significant and long-lasting changes in osteoblastogenesis *in vitro*.

Findings above led us to conclude that there potentially exists a preferential commitment of BMSCs extracted from SBA mice to the adipogenic pathway. Results show that adipogenesis and osteoblastogenesis seem to be affected by the severity of energy deficit and that at least an 18% weight loss is required to observe these alterations.

### Sirt1 Is Sustainably Altered in BMSCs From 18% and 24% Groups at 10 Weeks

To determine the potential molecular mechanism involved in the regulation of BMSC differentiation in SBA mice, the relative mRNA level of *Sirt1* was measured. Indeed, we recently demonstrated that 18% weight loss in mice is associated with the downregulation of the expression and activity of *Sirt1* and further, possibly leads to an increase in adipogenesis at the expense of osteoblastogenesis (26). In the current study, we hypothesised that *Sirt1* could be altered by the degree of severity of energy deficit, leading to an imbalance between osteoblastogenesis and adipogenesis. It is important to note that *Sirt1* is widely known for its pro-osteoblastic and anti-adipogenic effects (18–23). Indeed, *in vivo* activation of *Sirt1* restores bone mass, structure, and biomechanical properties in ovariectomised female mice (42) and it protects against age-associated bone loss in male mice (43). *In vitro* studies demonstrated that activation of *Sirt1* promotes osteoblastogenic differentiation at the expense of adipogenesis (18–21). Interestingly, our results demonstrated that BMSCs from SBA mice with moderate and severe weight loss (18% and 24%) presented a substantial low *Sirt1* mRNA level after 48 hours of culture and after 14 days of co-differentiation (**Figure 6**). These findings suggest that the decrease in *Sirt1* expression could be responsible for the observed increase in adipogenesis at the expense of osteoblastogenesis in these two groups.

Other studies have shown that CR can stimulate the expression and activity of *Sirt1* in various mammalian tissues, such as the liver or white adipose tissue (24, 25). It was also shown that CR decreases the mRNA level of *Sirt1* in the cerebellum and midbrain (44), suggesting tissue-dependent regulation of the expression. More recently, in contrast to our study, it was demonstrated that 12 months of 40% calorie restriction of male Sprague–Dawley rats (8 months old) induced an upregulation in protein expression of *Sirt1* in bone marrow fat compared to that in *ad libitum* animals (38). This discrepancy between our results and others from Cohen et al. (24) and (38) could be explained by changes in calorie or food restriction protocols, mouse strain or species, age, and sex. Indeed, twelve-month-old male rats were used in Cohen et al. studies and calorie restriction involved a daily food allotment of 60% of that eaten by the *ad libitum* animals immediately after weaning (24). In our study, eight-week-old female C57BL/6J mice were submitted to the 10-week SBA protocol. Furthermore, the weight loss of mice in each protocol of calorie restriction could also explain this discrepancy. Indeed, contrary to our protocol, which induces 12%, 18%, and 24% weight loss in mice, there is no information on weight loss in the study by Cohen et al. (24) and there was a 40% lower weight (vs CT) in the study by Duque et al. (38). Thus, the loss of Sirt1 seems to be closely associated to bone and BMSCs alterations and could explain the strong and long-lasting commitment of BMSCs towards the adipogenic pathway in SBA mice.

In our study, 18% is considered moderate weight loss, which is associated with a decrease in all parameters of body composition (low fat mass, tendency to low lean mass, low total body BMC, low femur and lumbar spine BMC) and cortical bone thickness. Furthermore, only in this weight loss category we found an upregulation of most of the adipocyte markers analyzed (*Pparg2, Lep*, and *Glut4*) and a downregulation of most of the osteoblast markers analyzed (*Runx2, Sp7*, and *Bglap*). In addition, this weight loss group displayed a strong decrease in *Sirt1* mRNA level. Considering these results, the rest of the study was dedicated to focusing on SBA 18% and investigating the effect of a short duration of SBA protocol (4 weeks) on body composition, bone architecture, BMA, BMSC differentiation and *Sirt1* expression.

### A Short Duration of Moderate Weight Loss Did Not Affect Bone Architecture, BMA, BMSC Differentiation or Sirt1 Expression

Four weeks of the SBA protocol induced only a decrease in fat mass (**Figure 7B**) and Tb.Th (**Figure 8B**) without affecting the other parameters of bone architecture and BMA (**Figure 8**). Selective change in Tb.Th does not seem to be associated with an overall bone alteration. Moreover, this short duration did not impact BMSC differentiation towards adipogenic lineage or osteoblastogenic lineage nor did it affect the unsaturation ratio of lipid droplets (**Figures 9**–**11**, **Supplementary Figure 2**). Interestingly, this lack of effects on bone and BMSCs differentiation is associated with a lack of effects on Sirt1 mRNA level. This strengthens the hypothesis of a direct involvement of Sirt1 alteration on BMSCs fate decision and bone. Altogether, these results suggest that in addition to the degree of severity, the duration of energy deficit could play an important role in regulating bone architecture, BMA, and BMSC differentiation, which requires long-term protocols to be altered. This line of inquiry will be required in the future to determine when the first alterations in bone, BMA, and *Sirt1* expression can occur in our SBA model.

## Conclusion

The present study demonstrated, for the first time, the impact of severity and length of energy deficit on bone and BMSCs differentiation capacity. The data from two groups (moderate and severe weight loss) support the hypothesis that there is a link between decrease in Sirt1 mRNA levels and alterations in bone and BMSCs differentiation capacity. A comparison of the 10-week SBA protocol to the 4-week SBA protocol strengthen this hypothesis. Finally, bone alterations appear to be totally disconnected from BMA changes in this model, probably because the increase in the number of BM pre-adipocytes, which takes place at the expense of osteoblastogenesis and does not result in an increase in mature adipocytes.

*In vivo* changes in BMSCs engagement in the adipogenic pathway remain to be specified using FACS analysis. Furthermore, the molecular mechanisms responsible for Sirt1 long lasting downregulation will be explored through the RNA sequencing approach.

## Data Availability Statement

The original contributions presented in the study are included in the article/**Supplementary Material**. Further inquiries can be directed to the corresponding authors.

## Ethics Statement

The animal study was reviewed and approved by the Committee on the Ethics of Animal Experiments (CEEA) of Nord-Pas de Calais, France (permit number: CEEA#2016070717275082).

## Author Contributions

CC, OG, and VA contributed to the conception and design of the work. VA carried out all the cell cultures experiments, qPCR and their analysis. DL and VG contributed to sample collection during mice sacrifice. JD and FM contributed to acquisition and analysis of the microCT data. GF performed the Raman analysis. VA, OG, and CC contributed to statistical analysis. VA and OG wrote the manuscript. CC and OG contributed to the review and editing the manuscript. All authors contributed to the article and approved the submitted version.

## Funding

VA is supported by Université du Littoral Côte d’Opale and Région Hauts-de-France.

## Conflict of Interest

The authors declare that the research was conducted in the absence of any commercial or financial relationships that could be construed as a potential conflict of interest.

## Publisher’s Note

All claims expressed in this article are solely those of the authors and do not necessarily represent those of their affiliated organizations, or those of the publisher, the editors and the reviewers. Any product that may be evaluated in this article, or claim that may be made by its manufacturer, is not guaranteed or endorsed by the publisher.
