# Supplementary material for: Severity Level and Duration of Energy Deficit in Mice Affect Bone Phenotype and Bone Marrow Stromal Cell Differentiation Capacity
Source: Front Endocrinol (Lausanne). 2022 Jun 6;13:880503. doi: 10.3389/fendo.2022.880503 (PMC9207532; doi:10.3389/fendo.2022.880503)
Supplement: Supplementary file 1 [file DataSheet_1.docx]

**Supplementary Data**

**RAMAN Spectroscopy of 10-week Day 14 Samples**

**Control**

**Supplementary Figure 1. Lipid unsaturation ratio in 10-week protocol groups**. Lipid droplet unsaturation ratio was compared between four SBA groups and the control. The results showed that there is a significant decrease in unsaturation ratio in weight loss groups when compared to the 0% group. The control group did not show a significant difference with weight loss groups (SBA 12%, 18%, 24%). Statistical analysis was performed using Mann–Whitney’s test.

**RAMAN Spectroscopy of 4-week Day 14 Samples**


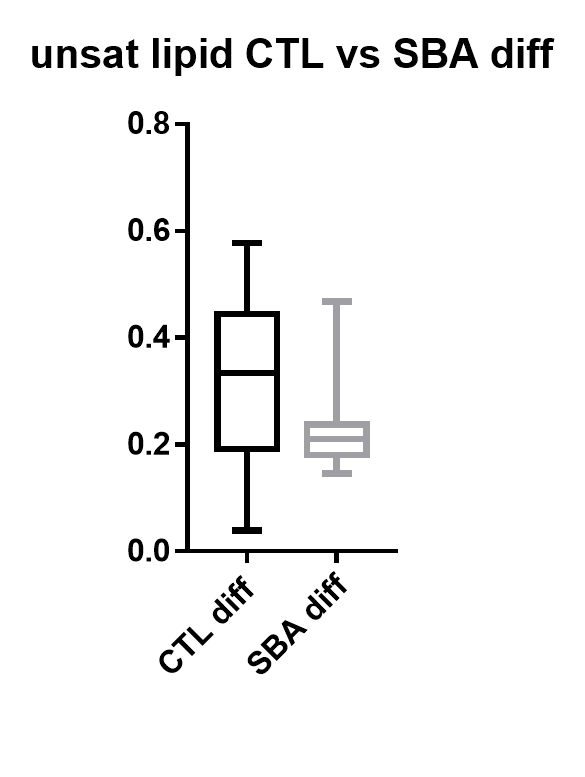


**Supplementary Figure 2. Lipid unsaturation ratio in 4-week protocol groups**. Lipid droplet unsaturation ratio was compared between the 18% SBA group and the control, and no difference was found between the two groups. Statistical analysis was performed using Mann–Whitney’s test.

**Unsaturation ratio**

**Control**

**18%**
